# Consistency analysis of two fingertip capillary blood sampling methods for complete blood count

**DOI:** 10.1038/s41598-024-64448-z

**Published:** 2024-07-01

**Authors:** Xie Hongxiang, Lv Shiyu, Zhang Yanying, Xu Wanju, Wang Sumei

**Affiliations:** 1 Laboratory Medicine Center, Department of Clinical Laboratory, Zhejiang Provincial People’s Hospital (Affiliated People’s Hospital), Hangzhou Medical College, No. 158 Shangtang Road, Hangzhou, 310014 Zhejiang People’s Republic of China; 2 Department of Clinical Laboratory Medicine, The First Affiliated Hospital of Shandong First Medical University & Shandong Provincial Qianfoshan Hospital, 16766 Jingshi Road, Jinan, 250014 Shandong People’s Republic of China; 3 Department of Clinical Laboratory, Zaozhuang Chest Hospital, Zaozhuang Tumor Hospital, Zaozhuang, Shandong People’s Republic of China

**Keywords:** Capillary blood, Blood collection tubes, Microhematocrit tubes, Complete blood count, Paediatric research, Laboratory techniques and procedures

## Abstract

This study was performed to analyze fingertip capillary blood sampling in pediatric patients using microcapillary blood collection tubes and microhematocrit tubes and to compare the blood cell analysis results obtained via these two blood collection methods. Finger capillary blood was collected from 110 outpatients using microcapillary blood collection tubes and microhematocrit tubes and complete blood count analysis was performed with a Sysmex XS-900i hematology analyzer and manual microscopy for blood cell morphology. Paired data was evaluated for agreement and bias using the microhematocrit samples as the reference group and the samples from the microcapillary blood collection tubes as the observation group. The two blood collection methods demonstrated good agreement for measuring red blood cell (RBC) parameters (i.e., RBC, Hb, Hct, MCV, MCH and MCHC), wherein the relative bias was > allowable total error (TEa) in 0.91%, 1.82%, 11.82%, 1.82%, 0.91% and 8.18% of the parameter measures, respectively. According to industry requirements, the proportion of samples meeting the acceptable bias level should be > 80%. Additionally, the estimated biases at each medical decision level were within clinically acceptable levels for RBC, Hb, Hct, and MCV. However, the proportion of WBC and PLT counts with relative bias > TEa was 25.45% and 35.45%, respectively. Furthermore, the relative bias of the WBC count at the medical decision level of 0.5 × 10^9^/L and that of the PLT counts at the medical decision levels of 10 × 10^9^/L and 50 × 10^9^/L were clinically significant. Bland–Altman analysis further showed a mean bias of 0.66 × 10^9^/L (95% LoA, − 0.79 to 2.11) for the WBC count and 39 × 10^9^/L (95% LoA, − 46 to 124) for the PLT count from the blood samples collected in the microcapillary blood collection tubes compared with the counts of those collected in the microhematocrit tubes. Neutrophil, monocyte, lymphocyte, eosinophil, and PLT counts increased significantly in the microcapillary blood collection tubes compared with those in the microhematocrit tubes, along with an elevated number of instrument false alarms (*P* < 0.05). The two capillary blood collection devices exhibit performance differences. Therefore, clinicians should pay attention to the variation in results caused by different blood collection methods.

## Introduction

Complete blood count (CBC) is a frequently used laboratory test. CBC can provide information on blood cell production, as well as offer data on the immune system via white blood cell (WBC) counts and determine the oxygen-carrying capacity of patients by assessing red blood cell (RBC), hemoglobin (Hb), and hematocrit (Hct) counts. Furthermore, these tests can aid in the diagnosis of anemia, certain cancers, infections, acute bleeding, allergies, and immune deficiencies, serving as practical tools for pediatricians^1^. Accurate and effective blood test results are highly dependent on high-quality samples. However, obtaining high-quality blood samples for CBC analysis in pediatric patients remains a challenge. Pediatric patients typically have poor compliance with spontaneous cooperation and possess slender blood vessels. Consequently, venous blood sampling is often accompanied by pain and mental stress. Moreover, venipuncture blood sampling in this patient population is not only extremely difficult but also carries potential risks. For example, deep venipuncture in children can lead to numerous adverse consequences, such as infection, cardiac arrest, bleeding tendency, and venous thrombosis^2,3^. Therefore, CBC tests in pediatric patients are frequently conducted using capillary blood collection.

Based on the available standards and recommendations such as the WHO Best Practices in Phlebotomy and the Clinical Laboratory Standards Institute (CLSI) GP42-A6, several national and international professional bodies, including those in China and Croatia, have issued corresponding guidance documents^4–7^. Capillary blood collection already occupies an indispensable position in pediatric clinical work. Compared with venous blood sampling, capillary blood collection has several contributing factors that make it prone to pre-analytical errors. Furthermore, most steps of these errors are not under the direct control of the laboratory^8^. Blood collection using microcapillary blood collection tubes or microhematocrit tubes are two capillary blood collection methods commonly used in clinical laboratories and recommended by various national capillary blood collection guidelines, including that of China^6,7^. Existing studies are primarily limited to comparisons between venous and capillary blood collection and have shown inconsistent results^9,10^. However, the effect of different capillary blood collection methods on CBC results has rarely been reported. In this study, we analyzed the consistency between the results obtained using microcapillary blood collection tubes and microhematocrit tubes for CBC analysis in daily practice, aiming to contribute to the clinical laboratory quality monitoring and interpretation of experimental results.

## Materials and methods

### Participant information

A total of 110 children (58 boys and 52 girls; minimum age: 6 months, maximum age: 14 years, mean age: 4.56 ± 3.3 years) who sought medical treatment at the outpatient department of the First Affiliated Hospital of Shandong First Medical University from January 2023 to December 2023 were randomly selected as participants. The study protocol was approved by the medical ethics committee of the hospital, and informed consent was obtained from the guardians of the children.

### Specimen collection and testing

**Figure 1:**
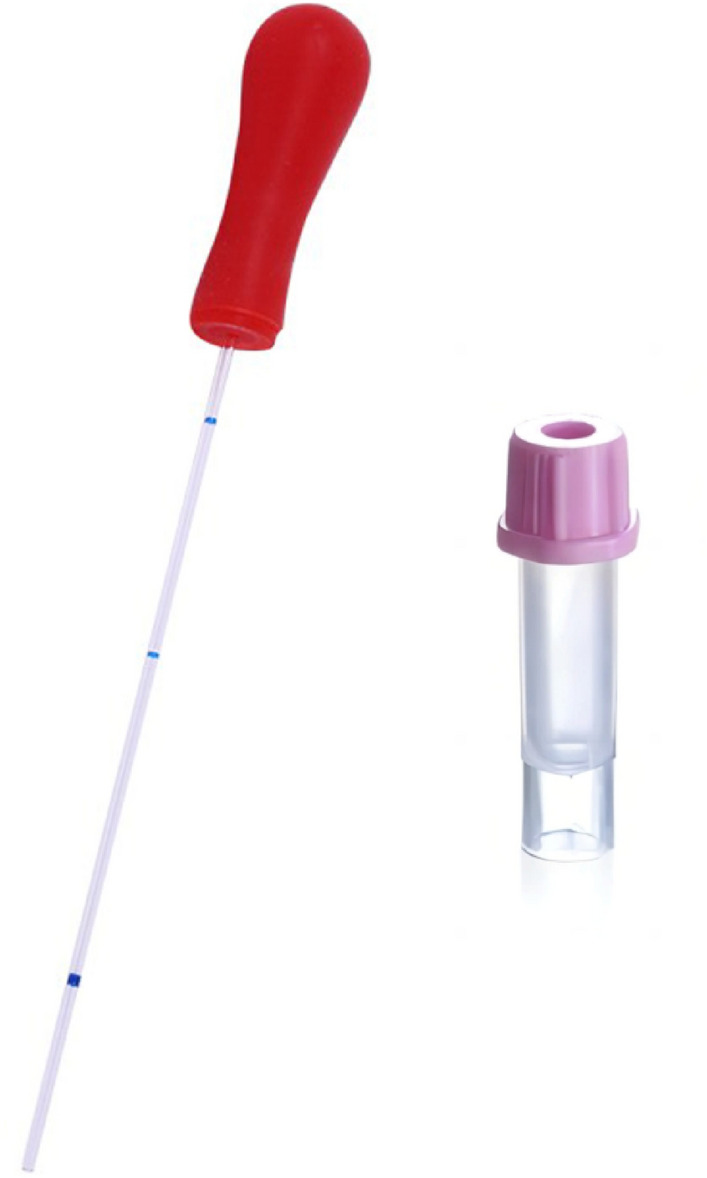
Schematic diagram of the two capillary blood collection methods. Left side: Microhematocrit tubes are disposable, hollow glass or plastic pipettes with quantitative identification. Blood can flow into the pipette through the siphoning effect. Right side: Microcapillary blood collection tubes are used to collect and store capillary blood samples after skin puncture. The tubes may contain anticoagulants according to the requirements of the CBC tests.

Capillary blood was collected by a trained phlebotomist. In this procedure, 70% isopropyl alcohol was first used to disinfect the inner side of the fingertip of the right ring finger of the child. After the natural drying of the fingertip, the skin was punctured using a pressure-type disposable capillary blood collection needles (BD Microtainer; Becton Dickinson, New Jersey). Next, the first drop was wiped, and the finger was gently pressed alternately to allow blood to flow out to form a blood drop. The blood drop was then randomly collected using a microhematocrit tube (Chengwu Medical Material Co., Ltd. Shandong) and a microcapillary blood collection tube (Gongdong Medical Technology Co., Ltd. Zhejiang). Blood was passed via a capillary into a microhematocrit tube and finally into a microcollection device containing EDTA-K2 anticoagulant for onboard testing. Microcapillary blood collection tubes mainly rely on the gravity-flow principle to collect blood samples. The two blood sampling methods are illustrated in Fig. 1. After gentle mixing, a Sysmex XS-900i automatic hematology analyzer and supporting reagents were used for the CBC analysis within 10 min. Background tests and internal quality control were conducted daily before the equipment was utilized for sample testing, along with regular inter-laboratory quality assessment.

### Method comparison and bias assessment

The bias of each paired sample was calculated according to the performance requirements of clinical hematology analysis in China National Accreditation Service for Conformity Assessment (CNAS) ISO15189—Guidance on the Application of Accreditation Criteria for the Medical Laboratory Quality and Competence^11,12^. The CBCs obtained from the blood samples collected in WHO-recommended microhematocrit tubes were used as reference values (X), and those acquired from the blood samples collected in microcapillary blood collection tubes were designated as measured values (Y). Further, % Bias = (measured value − reference value)/reference value * 100%. The allowable total error (TEa) for bias specified in the Clinical Laboratory Improvement Amendments of 1988 (CLIA' 88) was used as the acceptance limit (AL) to judge whether the bias was clinically significant^13^. Accordingly, comparability verification was deemed successful if more than 80% of the data met the ALs^12^. Moreover, based on Clinical and Laboratory Standards Institute (CLSI) guideline EP09—Measurement Procedure Comparison and Bias Estimation Using Patient Samples, the Passing–Bablok model was employed to fit the regression equation^14^. This step allowed the calculation of bias of the relevant parameters at medical decision levels (MDLs)^15^ during the testing of the samples collected by the two blood sampling methods. Lastly, Bland–Altman plots were utilized to determine the mean bias and 95% limits of agreement (LoA) between the two blood collection methods.

### Data statistics

Statistical analysis was performed using MedCalc 19.0 statistical software. The normality of data distribution was examined via the Kolmogorov–Smirnov test. Non-normally distributed data were presented as medians and percentiles, and comparisons between the two sets of samples were performed using the Wilcoxon rank sum test. The χ^2^ test was applied to compare the ratio of the two sample rates. Intraclass correlation coefficient (ICC) analysis was employed to evaluate the consistency of the paired data, with values > 0.80 indicating strong correlations. A *P*-value of < 0.05 was considered statistically significant. 

### Ethical approval

This study was conducted in accordance with the principles of the Helsinki Declaration, and it has received approval from the Shandong Provincial Qianfoshan Hospital Ethics Committee (No. 2022S484).

## Results

### Characteristic and comparability validation of paired data

ICC analysis showed strong correlations between the paired parameter measurements of leukocytes, erythrocytes, and platelets (PLT) in the CBC results of the samples acquired by the two blood collection methods. All relevant data are presented in Table 1. The comparability validation results demonstrated that the proportions of RBC, hemoglobin (Hb), hematocrit (Hct), mean corpuscular volume (MCV), mean corpuscular hemoglobin (MCH), and mean corpuscular hemoglobin concentration (MCHC) measures with relative bias > TEa in the paired samples from the two blood collection methods were 0.91%, 1.82%, 11.82%, 1.82%, 0.91%, and 8.18%, respectively. According to industry standards, the proportion of samples meeting acceptable bias should be > 80%. Thus, these findings suggest no clinically meaningful differences in the RBC-related parameters between the two blood collection methods, implying comparable results (Table 2). However, the proportion of WBC and PLT counts with relative bias > TEa in the paired samples was 25.45% and 35.45%, respectively, thereby exceeding the values specified by industry standards.

**Table 1:** Data distribution and intraclass correlation coefficient analysis of the complete blood count results according to the two blood collection methods.

Parameters	Microhematocrit tubes		Microcapillary blood collection tubes		ICC [95% CI]
	M (P25, P75) or $\overline{x }$±SD	Range (min–max)	M (P25, P75) or $\overline{x }$±SD	Range (min–max)	
WBC (× 10^9^/L)	6.47 (4.88, 8.88)	0.17–29.29	6.91 (5.62, 9.57)	0.43–34.99	0.9871 [0.9812–0.9911]
RBC (× 10^12^/L)	4.49 (4.09, 4.80)	0.75–5.84	4.47 (4.13, 4.79)	0.71–5.69	0.9914 [0.9875–0.9941]
Hb (g/L)	121.5 (112.0, 128.0)	24.0–156.0	121.0 (113.0, 130.3)	26.0–158.0	0.9885 [0.9832–0.9921]
Hct (L/L)	0.374 (0.351, 0.398)	0.076–0.469	0.365 (0.344, 0.389)	0.071–0.455	0.9828 [0.9750–0.9882]
MCV (fL)	84.0 (80.6, 86.6)	64.6–101.3	82.0 (78.9, 84.3)	58.0–100.0	0.9385 [0.9116–0.9574]
MCH (pg)	27.2 (26.3, 28.0)	14.5–32.0	27.4 (26.5, 28.1)	14.8–36.6	0.9630 [0.9464–0.9745]
MCHC (g/L)	324 (315, 331)	224–353	334 (327, 340)	256–366	0.8949 [0.8468–0.9280]
PLT (× 10^9^/L)	219 (162, 294)	1–911	262 (208, 332)	7–944	0.9419 [0.9164–0.9598]

ICC, intraclass correlation coefficient; CI, confidence interval; WBC, white blood cell; RBC, red blood cell; Hb, hemoglobin; Hct, hematocrit; MCV, mean cell volume; MCH, mean hemoglobin concentration; MCHC, mean cellular hemoglobin concentration; PLT, platelet.

**Table 2:** Relative deviation between paired measurements derived from microhematocrit tubes and microcapillary blood collection tubes.

Parameters	Mean relative bias (%)	Range of relative bias (%)	Allowable total error (CLIA' 88) (%)	Proportion of samples > TEa (CLIA' 88) (%)
WBC (× 10^9^/L)	11.53	− 12.07 to 152.94	± 15	25.45
RBC (× 10^12^/L)	− 0.07	− 18.43 to 5.95	± 6	0.91
Hb (g/L)	0.87	− 18.97 to 8.74	± 7	1.82
Hct (L/L)	− 2.58	− 22.87 to 4.61	± 6	11.82
MCV (fL)	− 2.40	− 11.67 to 8.61	± 10	1.82
MCH (pg)	0.89	− 3.63 to 14.38	± 9	0.91
MCHC (g/L)	3.44	− 1.85 to 15.82	± 7	8.18
PLT (× 10^9^/L)	25.80	− 24.05 to 154.17	± 25	35.45

Clinical Laboratory Improvement Amendments of 1988: CLIA’ 88; TEa: Allowable total error; WBC, white blood cell; RBC, red blood cell; Hb, hemoglobin concentration; Hct, hematocrit; MCV, mean cell volume; MCH, mean hemoglobin concentration; MCHC, mean cellular hemoglobin concentration; PLT, platelet.

### Bias analysis of the two blood collection methods

A detailed analysis of the bias at the MDL between the CBC results obtained using the two blood sampling methods was performed based on the CLSI-EP09c document guidelines. The regression equation and the results of each parameter are provided in Table 3. The findings revealed that the bias of RBC-related parameters (i.e., RBC, Hb, Hct, and MCV measures) at their respective MDLs was less than the TEa for both blood sampling methods, indicating that the results were comparable. However, the relative bias of the WBC count at the MDL of 0.5 × 10^9^/L was 46.15% and that of the PLT counts at the MDLs of 10 × 10^9^/L and 50 × 10^9^/L was 88.89% and 30.51%, respectively, in the paired samples. These three biases exceeded the TEa, suggesting clinically significant differences.

**Table 3:** Predicted bias of WBC, RBC, Hb, Hct, MCV, and PLT at different medical decision levels.

Parameters	Passing–Bablok regression equation	Slope 95% CI	Intercept 95% CI	MDL (*Xc*)	Predicted values (*Yc*)	(*Xc* + *Yc*)/2	Absolute bias	Relative bias (%)	ALs (%)	Clinical acceptability
WBC(×10^9^/L)	y = 0.283 + 1.043x	1.012–1.075	0.099–0.552	0.50	0.80	0.65	0.30	46.15	15	No
				3.00	3.41	3.21	0.41	12.77	15	Yes
				12.00	12.80	12.40	0.80	6.45	15	Yes
				30.00	31.57	30.79	1.57	5.10	15	Yes
RBC (× 10^12^/L)	y = 0.062 + 0.984x	0.950–1.008	− 0.044–0.221	3.50	3.51	3.51	0.01	0.28	6	Yes
				4.50	4.49	4.50	− 0.01	− 0.22	6	Yes
				6.80	6.75	6.78	− 0.05	− 0.73	6	Yes
Hb (g/L)	y = 2.261 + 0.989x	0.947–1.000	1.000–6.921	45	46.77	45.89	1.77	3.86	7	Yes
				105	106.11	105.56	1.11	1.05	7	Yes
				170	170.39	170.20	0.39	0.22	7	Yes
				230	229.73	229.87	− 0.27	− 0.12	7	Yes
Hct (L/L)	y = − 0.001 + 0.981x	0.939–1.017	− 0.013–0.014	0.140	0.136	0.138	− 0.004	− 2.899	6	Yes
				0.330	0.323	0.327	− 0.007	− 2.141	6	Yes
				0.560	0.548	0.554	− 0.012	− 2.166	6	Yes
				0.700	0.686	0.693	− 0.014	− 2.020	6	Yes
MCV (fL)	y = 0.637 + 0.971x	0.915–1.028	− 4.131–5.313	80.0	78.3	79.2	− 1.7	− 2.1	10	Yes
				100.0	97.7	98.9	− 2.3	− 2.3	10	Yes
PLT (× 10^9^/L)	y = 14.893 + 1.071x	1.021–1.135	3.365–25.110	10	26	18.00	16.00	88.89	25	No
				50	68	59.00	18.00	30.51	25	No
				100	122	111.00	22.00	19.82	25	Yes
				600	657	628.50	57.00	9.07	25	Yes
				1000	1086	1043.00	86.00	8.25	25	Yes

WBC, white blood cell; RBC, red blood cell; Hb, hemoglobin concentration; Hct, hematocrit; MCV, mean cell volume; PLT, platelet; CI, Confidence Interval; MDLs, medical decision levels; AL, acceptance limit.

**Figure 2:**
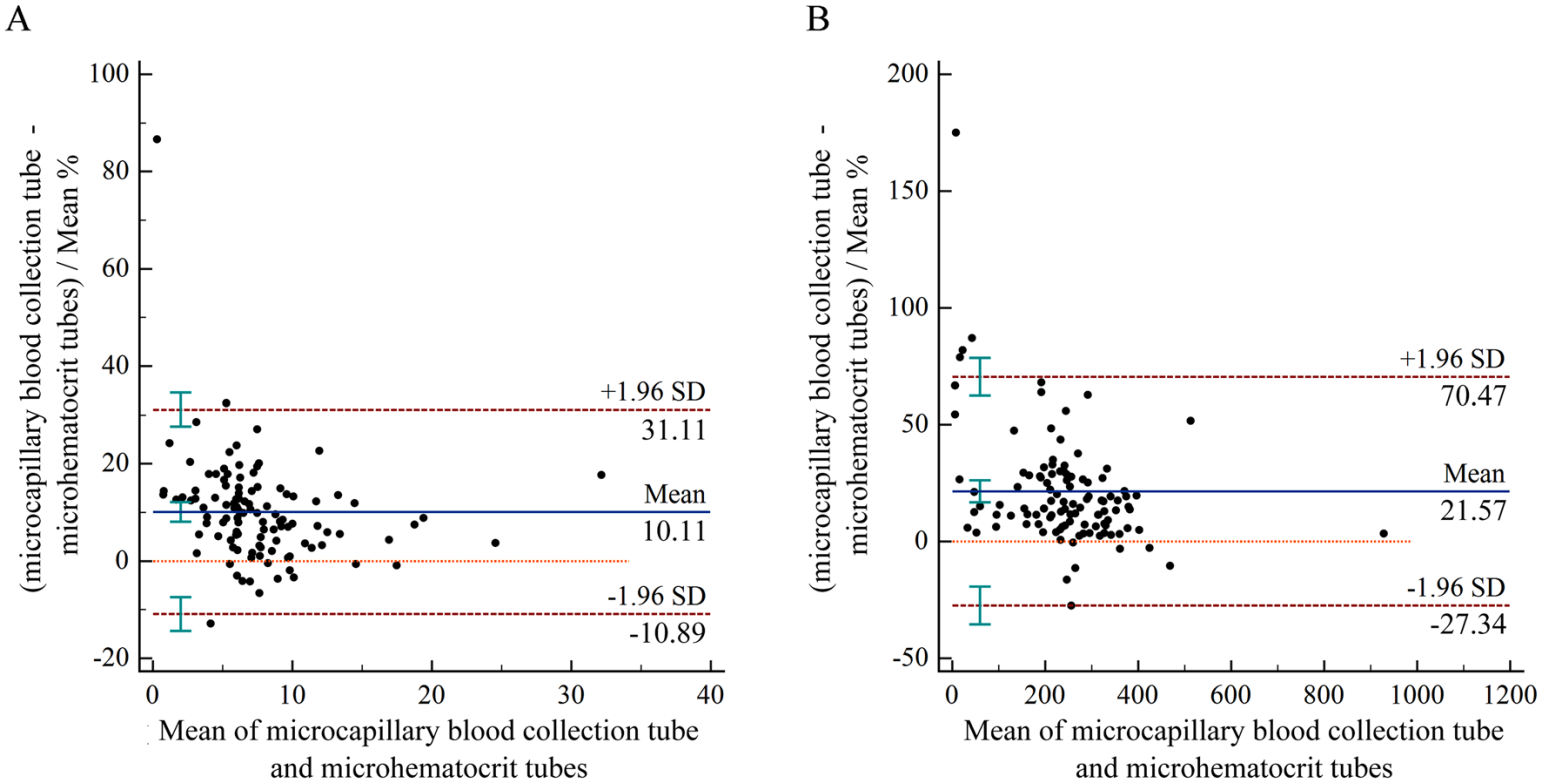
Results of the Bland–Altman analysis for comparing the WBC and PLT counts in the blood samples obtained using the microhematocrit tubes and microcapillary blood collection tubes, with the 95% LoA values. The scatter diagram displays the variations plotted against the averages of the two parameters, with the mean difference and the LoA represented by horizontal lines.

Additionally, Bland–Altman plots were employed to assess the average bias in the sample measurements for the two blood collection methods. As depicted in Fig. 2, the average bias in the WBC count for the blood samples collected using the microcapillary blood collection tubes was 0.66 × 10^9^/L (95% CI 0.52 × 10^9^/L to 0.80 × 10^9^/L) and the 95% LoA ranged from − 0.79 to 2.11, while the average relative bias was 10.11% (95% CI 8.09% to 12.14%) and the 95% LoA was − 10.89% to 31.11% compared with the microhematocrit tubes. In the case of PLT count, the mean bias was 39 × 10^9^/L (95% CI 31 × 10^9^/L to 48 × 10^9^/L) and the 95% LoA was − 46 to 124, whereas the mean relative bias was 21.57% (95% CI 16.85 to 26.28) and the 95% LoA ranged from − 27.34% to 70.47%.

### Comparison of test results for white blood cell differential

In the white blood cell differential analysis, the absolute counts of neutrophils, lymphocytes, monocytes, and eosinophils were significantly higher in the samples from the microcapillary blood collection tubes than in the microhematocrit tubes samples (*P* < 0.0001). However, no significant differences were detected in the neutrophil and monocyte percentages between the two blood sampling methods (*P* > 0.05). Furthermore, compared to the microhematocrit tube specimens, the specimens from the microcapillary blood collection tubes exhibited falsely decreased lymphocyte percentages and falsely increased eosinophil and basophil percentages (*P* < 0.0001), as displayed in Table 4.

**Table 4:** Comparison of white blood cell differentiation results of capillary blood samples collected using the two blood collection methods.

Parameters	Complete blood count (× 10^9^/L)				Classification (%)			
	Microhematocrit tubes	Microcapillary blood collection tubes	*Z* value	*P* value	Microhematocrit tubes	Microcapillary blood collection tubes	*Z* value	*P* value
Neutrophils	3.56 (2.05, 5.39)	3.78 (2.23, 6.05)	8.3623	< 0.0001	58.35 (38.40, 70.75)	58.10 (38.20, 70.08)	0.7878	0.4308
Monocytes	0.49 (0.33, 0.66)	0.55 (0.38, 0.73)	5.9744	< 0.0001	7.10 (5.08, 10.03)	7.30 (5.18, 9.85)	0.4129	0.6797
Lymphocytes	2.18 (1.48, 3.12)	2.04 (1.34, 2.91)	6.2704	< 0.0001	30.25 (20.78, 49.90)	29.30 (20.50, 49.05)	3.5462	0.0004
Eosinophils	0.06 (0.03, 0.15)	0.05 (0.01, 0.12)	6.4539	< 0.0001	0.85 (0.20, 1.93)	1.00 (0.48, 2.13)	3.7837	0.0002
Basophils	0.03 (0.02, 0.05)	0.01 (0.01, 0.02)	8.0696	< 0.0001	0.20 (0.10, 0.30)	0.50 (0.30, 0.80)	7.8273	< 0.0001

### Frequency comparison of instrument alerts for platelet aggregation and cell scatter plot differences in samples obtained via the two blood collection methods

**Figure 3:**
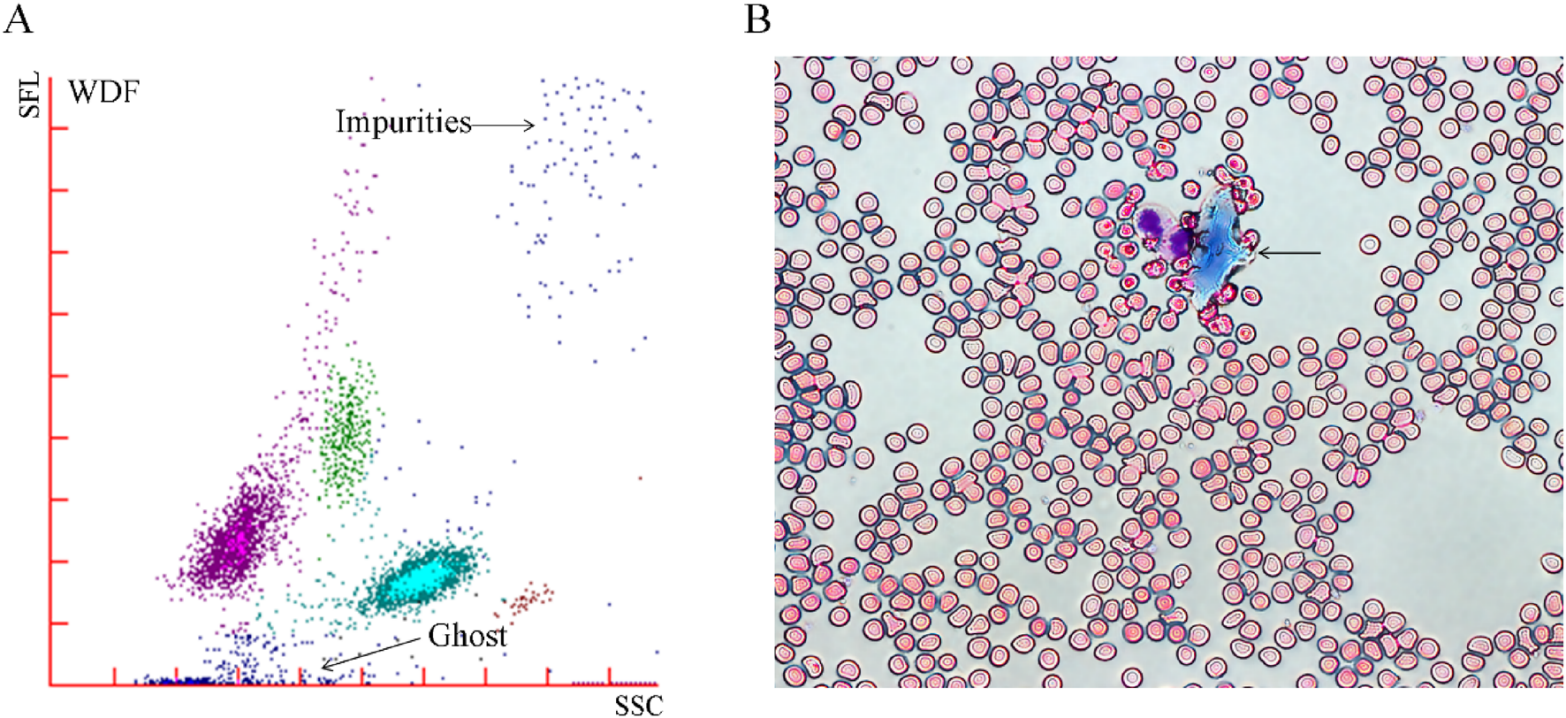
Impurities in blood samples collected using microcapillary blood collection tubes. (**A**) During the blood sampling process, impurities were detected in some blood samples. Additionally, prominent noise spots were observed in the scatter plot of the white blood cells in this blood sample, indicating the presence of impurities. Noise points caused by epithelial cells entering the sample during acquisition are in the upper right corner. Small impurities are lower near the x-axis (also called “Ghost”) and usually comprise broken blood cells. (**B**) Blood smear tests using the Wright-Giemsa staining method showed epithelial cells (arrows).

Instrument alert statistics revealed that 52 specimens collected using the microcapillary blood collection tubes triggered alarms for PLT aggregation, while only 25 specimens obtained via the microhematocrit tubes triggered these alarms. Moreover, there were 16 cases of alarm in both methods (Table 5). Additionally, smear microscopy indicated no cases of significant PLT aggregation. In the specimens collected from the microcapillary blood collection tubes, the WBC scatter plot analysis demonstrated prominent noise spots, which were further confirmed as keratinocytes through cell smear staining (Fig. 3). In contrast, the specimens obtained from the microhematocrit tubes presented no pronounced noise spots or keratinocyte debris.

**Table 5:** Comparison of instrument alarm frequency for platelet aggregation in capillary blood samples acquired using the two blood collection methods.

Method	Alarm	No alarm	χ^2^	*P*
Microcapillary blood collection tubes	52 (47.27%)	58 (52.73%)	14.57	< 0.001
Microhematocrit tubes	25 (22.73%)	85 (77.27%)

## Discussion

Pre-analytical quality assurance is the most critical aspect of clinical laboratory quality assurance. However, pre-analytical quality management is also the most latent and challenging component to control. According to reliable data, 60%–70% of the errors in clinical laboratories with well-established quality control systems occur during the pre-analytical phase^16^. Sample collection procedures are crucial for quality assurance, with specimen quality being a core issue in this step^17^. The accuracy of test results can be improved by making laboratory staff aware of the various biological factors that can affect test outcomes. In particular, pediatrics requires special attention because most blood specimens from children are collected utilizing capillary blood sampling methods. In this study, we compared the results of CBC tests performed using two commonly used capillary blood sampling methods, aiming to provide doctors with a basis for making diagnostic and therapeutic decisions.

The outermost layer of the epidermis is the stratum corneum, which is composed of multiple layers of flat, non-nucleated dead cells. The stratum corneum is continuously shed and renewed in cycles of 3 to 4 weeks and is eosinophilic homogeneous on light microscopy^18^. Our study found differences in skin or blood cell damage between the two blood collection methods. Blood sampling using microcapillary blood collection tube resulted in the sharp edge of the collection tube exerting forces on the keratinocytes. The dead keratinocytes, which are easily shed, form keratinocyte fragments of varying numbers and sizes and interfere with the results of some hematology analyzer tests.

In this study, RBC and PLT counts were detected via a Sysmex XS-900i hematology analyzer, which utilizes sheath flow and electrical resistance method. Our findings demonstrated a strong correlation between the test results of the RBC parameters, such as RBC, Hb, Hct, and MCV, in the two capillary blood sampling methods, with the test result differences exhibiting no clinical significance. This observation suggests that the impact on the RBC test results was minimal, possibly due to the relatively smaller number of epidermal keratin fragments contaminating the specimens compared with the RBC numbers. In contrast, the PLT count performed by the Sysmex XS-900i could have easily misidentified epidermal keratin fragments as PLT because of their similar size, thereby leading to falsely elevated PLT test results in the blood specimens collected using microcapillary blood collection tubes. Furthermore, the PLT counts at the MDL of 10 × 10^9^/L and 50 × 10^9^/L exhibited clinically significant bias between the two blood collection methods. Simultaneously, the false alarm rate for “PLT aggregation” in the samples procured using microcapillary blood collection tubes was significantly higher than that in the samples obtained using microhematocrit tubes, potentially due to the presence of epidermal keratinous debris contaminants. Microhematocrit tubes suck blood by employing the siphon principle, thus avoiding scraping the epidermal stratum corneum. Microscopic examination of the 110 specimens showed no apparent epidermal keratin fragments, and the false alarms were significantly reduced.

The Sysmex XS-900i hematology analyzer utilizes semiconductor-based laser light scattering and cytochemical staining to accurately classify WBCs with a modified flow analysis device. Our study revealed that 25.45% of the WBC counts in the paired samples from the two blood sampling methods had a bias greater than the TEa and higher than industry requirements. This outcome may be attributed to the limited strength of the hemolytic agent, resulting in the incomplete dissolution of epidermal keratinous debris in the specimens. The incomplete dissolution leads to the scattering of this debris in the scatter-plot of different cells according to their size after the staining process, thereby falsely increasing the cell counts of each cell population and ultimately causing a statistical difference in the percentage of some cells. The time from blood collection to detection was an important factor affecting the accuracy of CBC results ^19^. The WBC can be changed due to the time from blood collection to performing CBC tests. Especially for platelets, the appearance of aggregates depends on the time between blood collection and CBC tests^20^. In this study, after the patient samples were collected, the professionals analyzed them immediately, which reduced the influence of time on the test results.

Nevertheless, this study has a few limitations that should be considered. Given the different detection principles employed in various five-part differential hematology analyzers, the interference of epidermal keratinous fragments on the detection results may also vary. Additionally, we only compared two brands of microcapillary blood collection tubes and microhematocrit tubes in this study. The structural design and additives of capillary blood collection tubes can directly affect the accuracy of CBC results^21^.

In summary, blood collected using microcapillary blood collection tubes tends to be mixed with epidermal keratin debris that interferes with the WBC differential and PLT count on the Sysmex XS-900i hematology analyzer and causes increased instrument false alarms. Our study results suggest that microhematocrit tubes are significantly better than microcapillary blood collection tubes for blood collection. Therefore, different laboratories should evaluate the reliability of these results by using several different brands of capillary blood collection tubes.

## Data Availability

The corresponding author has all the data associated with this study. The data supporting the findings of this study are available from the corresponding author upon reasonable request.
